# *CYP7A1*, *NPC1L1*, *ABCB1*, and *CD36* Polymorphisms Are Associated with Increased Serum Coenzyme Q_10_ after Long-Term Supplementation in Women

**DOI:** 10.3390/antiox10030431

**Published:** 2021-03-11

**Authors:** Michiyo Takahashi, Mayumi Nagata, Tetsu Kinoshita, Takehiko Kaneko, Toshikazu Suzuki

**Affiliations:** 1 Graduate School of Human Ecology, Wayo Women’s University, 2-3-1 Konodai, Ichikawa, Chiba 272-8533, Japan; michiyo.takahashi1202@gmail.com (M.T.); t-kaneko@wayo.ac.jp (T.K.); 2 Department of Health and Nutrition, Wayo Women’s University, 2-3-1 Konodai, Ichikawa, Chiba 272-8533, Japan; k11.clover13@gmail.com; 3 Special Course of Food and Health Science, Department of Bioscience Graduate School of Agriculture, Ehime University, 3-5-7 Tarumi, Matsuyama, Ehime 790-8566, Japan; tetsu@shin-science.co.jp; 4 Institute of Community Life Sciences Co., Ltd., 1383-2 Hiramachi, Matsuyama, Ehime 791-0243, Japan

**Keywords:** bioavailability, cholesterol, coenzyme Q_10_, single nucleotide polymorphisms

## Abstract

Coenzyme Q_10_ (CoQ_10_), an essential component for energy production that exhibits antioxidant activity, is considered a health-supporting and antiaging supplement. However, intervention-controlled studies have provided variable results on CoQ_10_ supplementation benefits, which may be attributed to individual CoQ_10_ bioavailability differences. This study aimed to investigate the relationship between genetic polymorphisms and CoQ_10_ serum levels after long-term supplementation. CoQ_10_ levels at baseline and after one year of supplementation (150 mg) were determined, and eight single nucleotide polymorphisms (SNPs) in cholesterol metabolism and CoQ_10_ absorption, efflux, and cellular uptake related genes were assessed. Rs2032582 (*ABCB1*) and rs1761667 (*CD36*) were significantly associated with a higher increase in CoQ_10_ levels in women. In addition, in women, rs3808607 (*CYP7A1*) and rs2072183 (*NPC1L1*) were significantly associated with a higher increase in CoQ_10_ per total cholesterol levels. Subgroup analyses showed that these four SNPs were useful for classifying high- or low-responder to CoQ_10_ bioavailability after long-term supplementation among women, but not in men. On the other hand, in men, no SNP was found to be significantly associated with increased serum CoQ_10_. These results collectively provide novel evidence on the relationship between genetics and CoQ_10_ bioavailability after long-term supplementation, which may help understand and assess CoQ_10_ supplementation effects, at least in women.

## 1. Introduction

Coenzyme Q_10_ (CoQ_10_) is a vitamin-like molecule that is involved in ATP synthesis in mitochondria and is a crucial antioxidant that protects against oxidative damages of cell membranes and lipoproteins [1,2,3]. CoQ_10_ supplementation slowed aging and decreased protein, lipid, and DNA oxidative damage in the senescence-accelerated mouse prone 1 mice [4,5]. Similarly, reduced DNA oxidative damage by CoQ_10_ supplementation was observed in male Wistar rats [6]. In addition, CoQ_10_ supplementation ameliorated fatigue and promoted exercise performance, possibly by combining enhanced energy production in mitochondria and enhanced antioxidant protection, in male Institute of Cancer Research mice [7]. Human interventional studies also suggest the beneficial effects of CoQ_10_, including anti-inflammatory activities, prevention or improvement of degenerative disorders affecting longevity [8], enhanced vitality of patients undergoing medical treatment as well as on elderly residents of nursing homes [9,10,11], and alleviation of fatigue in patients with chronic fatigue syndrome [12,13]. Hence, CoQ_10_ is considered a health-supporting and antiaging supplement. However, meta-analyses of these studies provided inconsistent results related to the effects of CoQ_10_ supplementation [14,15,16,17] and even failed to show any beneficial effect of CoQ_10_ supplementation [14,15,16]. Hence, further studies are required to produce substantial clinical evidence supporting the benefits of CoQ_10_ supplementation [18].

One of the challenges to generate reliable evidence for the beneficial effects of CoQ_10_ supplementation is its low bioavailability due to poor water solubility and high molecular weight. Several types of CoQ_10_ delivery systems and a stable capsule-based product containing ubiquinol (the reduced form of CoQ_10_) were developed to increase the bioavailability of supplemental CoQ_10_ [19,20,21,22,23,24]. However, considerable variation and high standard derivations were reported for the single-dose pharmacokinetics and bioavailability of CoQ_10_ [22,23]. A large variance in the serum CoQ_10_ levels was also observed after long-term CoQ_10_ supplementation [25,26], suggesting that such variances may be another reason to affect the accurate recognition of the beneficial effects of CoQ_10_.

The absorption of supplemental CoQ_10_ into the bloodstream [27] starts with enterocytes absorbing CoQ_10_ in the intestine with the aid of carrier molecules/proteins on the cell membrane. The cholesterol transporter Niemann–Pick C1-like 1 (NPC1L1) protein is a candidate molecule to contribute to the absorption process [28,29]. Next, the CoQ_10_ molecules are incorporated into chylomicrons, which circulate in the lymph and blood, carrying the CoQ_10_ to the liver, where it is attached to the low-density and very-low-density lipoproteins. Then, the CoQ_10_ returns to the bloodstream as a lipoprotein complex. Hence, serum CoQ_10_ levels correlate strongly and positively with serum cholesterol levels [30]. Some exogenous CoQ_10_ can also be incorporated into cells, although the exact mechanism remains unclear [31]. Recently, Anderson et al. reported that the scavenger receptor CD36 drives the uptake of CoQ_10_ in brown adipose tissue, where it is required for normal physiological function [32]. In turn, the ATP-binding cassette subfamily B member 1 (ABCB1) expressed in enterocytes may excrete the absorbed CoQ_10_ into the intestinal lumen [33]. Thus, several proteins involved in CoQ_10_ absorption/incorporation and cholesterol metabolism adjust the individual serum CoQ_10_ levels during supplementation.

The involvement of genetic polymorphism in the pharmacokinetic profile, bioavailability, and metabolism of various drugs and nutrients has been reported in the last two decades. For example, single nucleotide polymorphisms (SNPs) and haplotypes of *ABCB1* are associated with altered drug disposition, drug response, and toxicity [34]. *CD36* SNPs and haplotypes are associated with serum LDL and total cholesterol levels [35]. Recently, Tomei et al. reported that rs731236 in the vitamin D receptor (*VDR*) gene and rs7116978 in the cytochrome p450 family 2 subfamily R member 1 *(CYP2R1*) gene were associated with serum 25-hydroxyvitamin D levels, the primary circulating form of vitamin D, after 12 weeks of vitamin D supplementation [36]. Only a few studies have reported an association between genetic polymorphism and basal serum CoQ_10_ status in humans [37,38] and increased serum CoQ_10_ levels after the two-week supplementation [38], but there is no study reporting the association after long-term supplementation.

In this study, the impact of polymorphisms in genes potentially involved in CoQ_10_ bioavailability on serum CoQ_10_ levels after long-term supplementation was evaluated in volunteers of *the Ubiquinol Health Examination*, a prospective intervention trial held in the Ehime Prefecture, Japan [26,39]. The SNPs were assessed in the following genes involved in the cholesterol metabolism: sterol regulatory element-binding protein 2 (*SREBP2*), 3-hydroxy-3-methylglutaryl–coenzyme A reductase (*HMGCR*), apolipoprotein B (*APOB*), cytochrome P450 family 7 subfamily A member 1 (*CYP7A1*), and CoQ_10_ absorption (*NPC1L1*), excretion (*ABCB1*), and cellular uptake (*CD36*). We further investigated whether genotyping of SNPs in the above genes is useful for classifying high- or low-responder to CoQ_10_ bioavailability after long-term supplementation.

## 2. Materials and Methods

### 2.1. CoQ_10_ Supplementation

Two types of reduced CoQ_10_ (ubiquinol) supplements (soft-encapsulated and granulated form) were provided by the Kaneka Co. (Osaka, Japan). The soft capsule Kaneka QH^TM^ contains reduced CoQ_10_ (50 mg per capsule), rapeseed oil, beeswax, soy lecithin, polyglycerol esters of fatty acids, gelatin, glycerol, and caramel. The granulated form P30 contains 30% (*w/w*) reduced CoQ_10_ (150 mg per sachet), dextrin, gum Arabic, and L-ascorbate. The participants could choose either the capsule or the granulated form of the supplement, taking three capsules or one sachet per day, respectively, corresponding to a daily dose of 150 mg CoQ_10_ during the intervention trial. The participants also could switch between supplement types every three months.

### 2.2. Study Design

**Figure 1:**
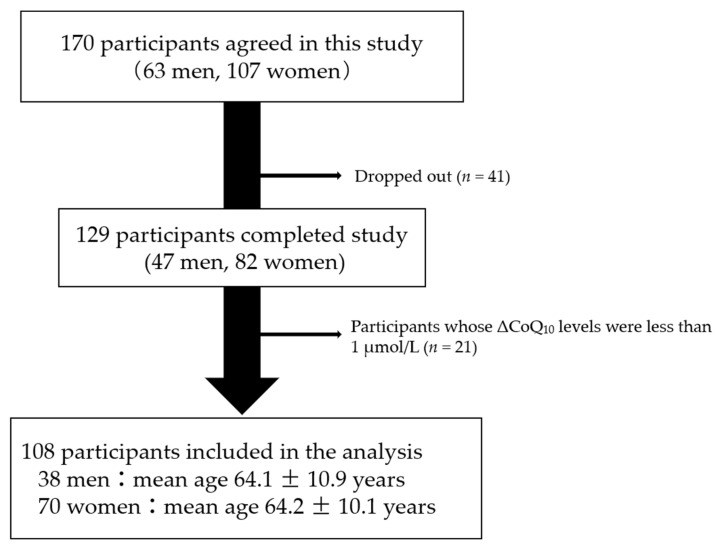
Flow chart describing the study design.

A total of 170 participants who had been participating in the “*Verification of health enhancement and QOL improvement effect by continuous ubiquinol ingestion (Ubiquinol Health Examination)*” study (UMIN000012612) [26,39] were recruited (Figure 1). All participants were residents of Kamijima-town of Ehime Prefecture, Japan. Kamijima consists of 25 small islands located in the Seto Inland Sea. Since Kamijima is a super-aging region (population aging rate is more than 40%), the authors considered Kamijima was appropriate to evaluate the effects of antiaging materials such as CoQ_10_. The participants took 150 mg per day of reduced CoQ_10_ in the postprandial state (after breakfast or lunch) from November 2016 to November 2017. First blood samples were collected at the time of enrollment in the Kamijima Ubiquinol Health Examination study (from November 2013 to November 2016), which served as a baseline, and another sample was collected in November 2017, after 1 year of 150 mg reduced CoQ_10_ supplementation. All participants provided informed consent before they participated in this study. This study was conducted in accordance with the Declaration of Helsinki, and it was approved by the Wayo Women’s University Human Research Ethics Committee (No. 1614). Participants were excluded from the analysis if the increased serum CoQ_10_ levels were less than 1 μmol/L.

### 2.3. Measurements of Serum CoQ_10_ and Total Cholesterol

A non-fasting blood sample was drawn between 9:00 and 15:00 on the test day when it was convenient for each participant. Blood serum was prepared by centrifugation at 3000 rpm for 10 min. For quantification of serum CoQ_10_ levels, 0.1 mL of serum was mixed with 0.7 mL 2-propanol and stored at −80 ℃ until just before the analysis. Quantitative analysis of serum CoQ_10_ concentration was performed by Kaneka Techno Research Co. (Osaka, Japan) using liquid chromatography with tandem mass spectrometry (LC/MS/MS), as described previously [40,41].

Serum total cholesterol (TC) levels were determined enzymatically (cholesterol esterase-cholesterol oxidase-peroxidase system) at Shikoku Chuken, Inc. (Kagawa, Japan).

### 2.4. Polymerase Chain Reaction (PCR)-Restriction Fragment Length Polymorphism (RFLP) Genotyping

Genomic DNA was isolated from the whole blood samples using the Maxwell RSC Blood DNA kit and the Maxwell RSC Instrument (Promega Corporation. Madison, WI, USA), according to the manufacturer’s instructions. DNA concentrations were determined using Qubit dsDNA BR assay kit (Thermo Fisher Scientific, Waltham, MA, USA) and diluted to a working concentration of 10 ng/µL. The PCR mixtures (20 µL) consisted of 0.2 µmol/L forward and reverse primer pairs (Table 1), 0.2 mmol/L dNTP mixtures, 1 ng/µL template DNA, and 0.01 U/µL Hot-start gene *Taq* NT DNA polymerase (Nippon Gene Co., Toyama, Japan) in (1×) HS Gene *Taq* NT buffer. The PCR amplification was performed on a TaKaRa PCR thermal cycler dice gradient (TaKaRa Bio, Shiga, Japan), and the amplification conditions were as follows: an initial activation step of 5 min at 95 ℃, 40 cycles of 30 s at 95 ℃ (denaturation), 30 s at 60 ℃ (annealing), and 1 min at 72 ℃ (extension), followed by a final extension for 10 min at 72 ℃. Amplification was confirmed on a 2% agarose gel electrophoresis in a tris-borate-EDTA buffer. Then, residual 10 µL of the PCR products were directly digested overnight with an appropriate restriction enzyme (Table 1). The digested DNA fragments were electrophoretically separated on a 2% agarose 21 (Nippon Gene Co.) gel. The genotypes detected by PCR-RFLP were confirmed by direct DNA sequencing of the PCR products using ABI PRISM 3100 genetic analyzer (Thermo Fisher Scientific) except for rs133291 in *SREBP2*.

### 2.5. Data Analysis

Microsoft Excel 2016 (Microsoft Corporation, Redmond, WA, USA) was used for statistical analyses. The increased serum CoQ_10_ (ΔCoQ_10_) and cholesterol-adjusted CoQ_10_ (serum CoQ_10_ per TC, Δ[CoQ_10_/TC]) levels were calculated by subtracting the baseline value from the value after supplementation. We first classified the participants into two groups by the ΔCoQ_10_ and Δ[CoQ_10_/TC] levels to assess the association of each SNP with the response to CoQ_10_ supplementation. The participants, whose levels were not less than the average value, were grouped as high-responders (HR), while others were grouped as low-responders (LR). A chi-squared test was performed to investigate the association and to confirm Hardy–Weinberg equilibrium (HWE) of SNP genotypes. All statistical assessments were two-tailed and considered significant at *p* < 0.05. All HWE *p*-values were more than 0.9, demonstrating that all SNPs meet HWE criteria. Welch’s *t*-test was performed when comparing the ΔCoQ_10_ and Δ[CoQ_10_/TC] values after separating the participants by genotype into two groups.

## 3. Results

Although 170 participants agreed to participate in this study, 41 dropped out during the intervention. Moreover, 21 participants whose serum ΔCoQ_10_ levels were less than 1 μmol/L were also excluded from the analysis, as they seemed not to follow the daily CoQ_10_ supplement program. Therefore, data from 108 participants were analyzed (Figure 1). The baseline characteristics of the participants are listed in Table 2.

**Figure 2:**
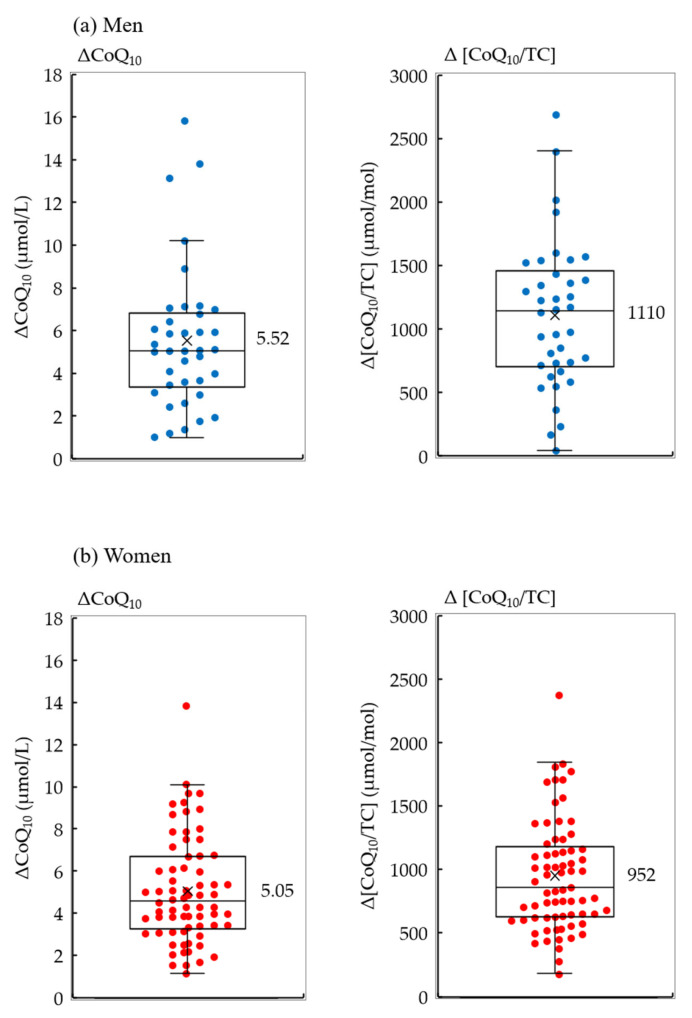
Serum ΔCoQ_10_ and Δ[CoQ_10_/TC] levels after 1 year of supplementation. Beeswarm box-plots of ΔCoQ_10_ and Δ[CoQ_10_/TC] for (**a**) men and (**b**) women. The bottom of the box is the 25th percentile, the line that intersects the box is the median, the multiplication sign within the box is the mean, and the top of the box is the 75th percentile. Whiskers above and below the box represent the 10th and 90th percentiles, and the points above and below the whiskers indicate the outliers. Differences between the two groups were analyzed using Welch’s *t*-tests (*p* < 0.05). ΔCoQ_10_, increased serum coenzyme Q_10_; Δ[CoQ_10_/TC], cholesterol-adjusted increased serum CoQ_10_.

The ΔCoQ_10_ and Δ[CoQ_10_/TC] levels showed wide interindividual variability (Figure 2), as reported previously [26]. The mean values of the ΔCoQ_10_ in men and women were 5.52 and 5.05 μmol/L, while those of the Δ[CoQ_10_/TC] were 1110 and 952 μmol/mol, respectively. To investigate the involvement of genetic variations in the ΔCoQ_10_ and Δ[CoQ_10_/TC], eight SNPs of seven genes were evaluated (Table 1), and PCR-RFLP was performed for genotyping them in each participant (Figure S1). The genotypes identified by PCR-RFLP were also confirmed by direct sequencing, except for *SREBP2* (Figure S1).

To assess the association between each SNP and the response to CoQ_10_ supplementation, the participants were divided into two groups (HR and LR) according to the average ΔCoQ_10_ and Δ[CoQ_10_/TC] levels. Among women, rs2032582 (*ABCB1*) and rs1761667 (*CD36*) were associated significantly with the HR/LR classification of ΔCoQ_10_ (chi-squared test, *p* = 0.049 and *p* = 0.022, respectively; Table 3). In addition, in women, rs3808607 (*CYP7A1*) and rs2072183 (*NPC1L1*) showed a significant association with the HR/LR classification of Δ[CoQ_10_/TC] (chi-squared test, *p* = 0.037 and *p* = 0.027, respectively; Table 3). In contrast, none of the SNPs were associated with the HR/LR classification of ΔCoQ_10_ or Δ[CoQ_10_/TC] in men.

**Figure 3:**
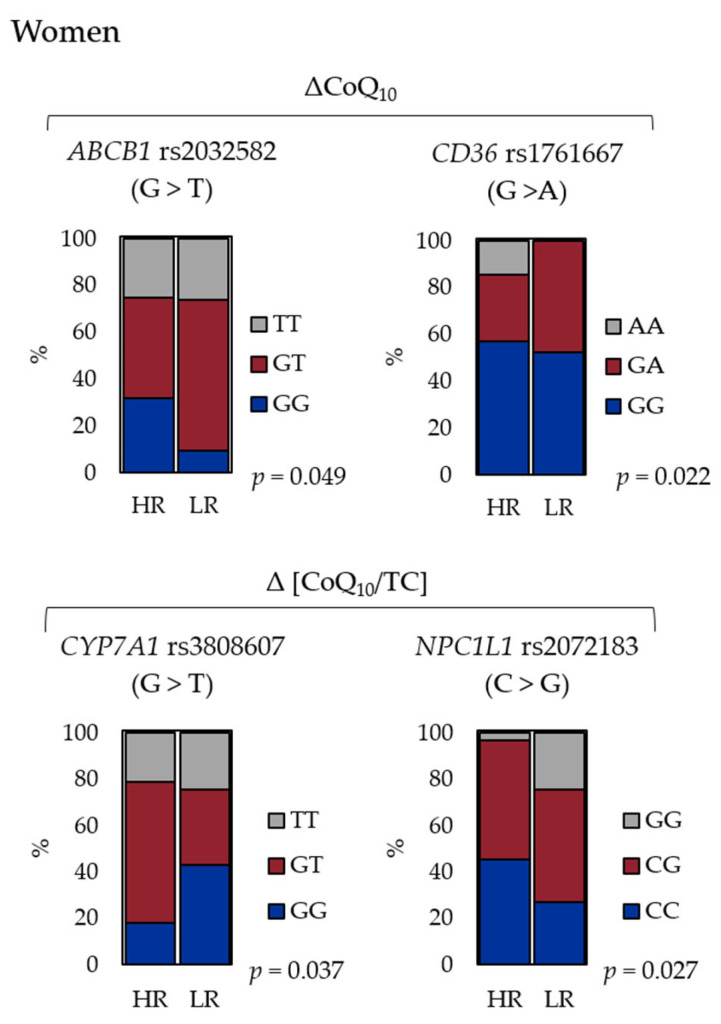
Genotypic frequency of the SNPs identified as correlated with the high-responders/low-responder (HR/LR) classification. The frequency of the genotypes of *ABCB1* rs2032582 and *CD36* rs1761667 associated with the ΔCoQ_10_, and *CYP7A1* rs3808607 and *NPC1L1* rs2072183 with the Δ[CoQ_10_/TC] in women were shown. ΔCoQ_10_, increased serum coenzyme Q_10_; Δ[CoQ_10_/TC], cholesterol-adjusted increased serum CoQ_10_; HR, high-responder; LR, low-responder.

Next, the SNPs identified with significantly low *p*-values of ΔCoQ_10_ and Δ[CoQ_10_/TC] were further evaluated in women. Figure 3 shows the frequency of the different genotypes according to the HR/LR classification in the women. The rs2032582 GG and rs1761667 AA were associated with a “ΔCoQ_10_ HR” status, and rs3808607 GT/TT and rs2072183 CC were associated with a “Δ[CoQ_10_/TC] HR” status.

**Figure 4:**
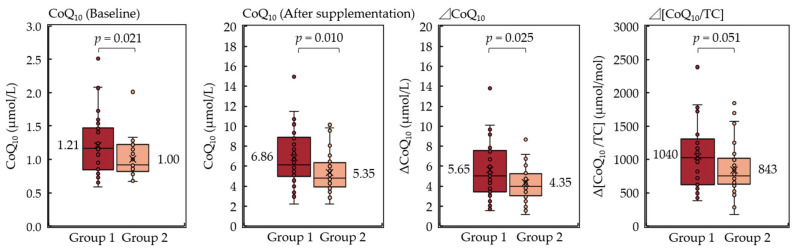
Box plots showing the serum CoQ_10_ levels at baseline and after 1 year of supplementation, ΔCoQ_10_, and Δ[CoQ_10_/TC] in women. The bottom of the box is the 25th percentile, the line that intersects the box is the median, the multiplication sign within the box is the mean, and the top of the box is the 75th percentile. Whiskers above and below the box represent the 10th and 90th percentiles, and the points above and below the whiskers indicate the outliers. Differences between the two groups were analyzed using Welch’s *t*-tests (*p* < 0.05). ΔCoQ_10_, serum coenzyme Q_10_; Δ[CoQ_10_/TC], cholesterol-adjusted serum CoQ_10_.

Next, the women participants were divided into two groups by combining the four identified SNPs in women, and their serum levels of CoQ_10_, ΔCoQ_10_, and Δ[CoQ_10_/TC] were compared (Figure 4). Group 1 consisted of the participants who had four or more of rs3808607 T, rs2072183 C, rs2032582 G, and rs1761667 A alleles, whereas group 2 consisted of the participants who had three or less of these alleles. The serum CoQ_10_ levels before and after supplementation and the ΔCoQ_10_ of group 1 were significantly higher than that of group 2 (Welch’s *t*-test, *p* = 0.021, *p* = 0.010, and *p* = 0.025, respectively; Figure 4). The Δ[CoQ_10_/TC] was of borderline significance between the two groups (*p* = 0.051), but group 1 showed a tendency for higher values than group 2. Each group of women was further subdivided into HR or LR according to ΔCoQ_10_ and Δ[CoQ_10_/TC] average values to assess potential associations with the response to CoQ_10_ supplementation (Table 4). The group with the above indicated four SNPs showed a significant association with the HR/LR classification of the Δ[CoQ_10_/TC] (chi-squared test, *p* = 0.003). The association between the group and the HR/LR classification of the ΔCoQ_10_ was borderline significant (chi-squared test, *p* = 0.063). A similar subgroup analysis was performed with the above four SNPs in the men, but no statistical differences were detected between the two groups (Figure S2A). These results suggest that genotyping of the above four SNPs is useful for predicting the HR/LR classification of the serum ΔCoQ_10_ and Δ[CoQ_10_/TC] levels prior to the long-term CoQ_10_ supplementation at least in women.

## 4. Discussion

The present study successfully identified four SNPs involved in the large individual variability in serum ΔCoQ_10_ and Δ[CoQ_10_/TC] levels after long-term supplementation in women. These SNPs are present in genes involved in cholesterol metabolism (*CYP7A1* rs3808607) [42], CoQ_10_ absorption at the intestinal epithelium (*NPC1L1* rs2072183) [29,43], CoQ_10_ efflux (*ABCB1* rs2032582) [33], and cellular CoQ_10_ uptake (*CD36* rs1761667) [32] (Table 3). Hence, classification by a combination of four SNPs may enable predicting HR or LR of CoQ_10_ supplementation in women (Table 4).

*CYP7A1* encodes the rate-limiting enzyme, cholesterol 7α-hydroxylase, which regulates bile acids synthesis. Participants with rs3808607 GG show lower cholesterol levels with a higher level of bile acid synthesis after ingesting high-molecular-weight β-glucan [44]. Moreover, the GT/TT individuals have higher serum cholesterol levels than those harboring the GG genotype. NPC1L1 plays a pivotal role in intestinal cholesterol absorption in addition to its involvement in CoQ_10_ absorption. The hepatic expression of *NPC1L1* was higher in rs2072183 CC individuals with gallstone disease than in those with CG/GG [45]. *ABCB1* encodes the *P-*glycoprotein pump involved in drug efflux. In patients with colorectal cancer, rs2032582 GG genotype correlates with the highest *P-*glycoprotein expression in the tumor tissue [46]. In accordance with the *ABCB1* expression, its rs2032582 polymorphism was associated with irinotecan-induced severe mucositis in metastatic colorectal cancer patients [47]. CD36 is a multi-ligand scavenger receptor whose primary function is to take up fatty acids and oxidized lipoproteins into cells [48]. The rs1761667 A allele reduces *CD36* expression in monocytes [49] and lowers sensitivity to fatty acid molecules [50]. Based on these reports, it is reasonable to hypothesize that the T allele of rs3808607 is involved in raising serum CoQ_10_ levels accompanied by serum TC levels, while the C allele of rs2072183 is related to increased CoQ_10_ transport from the intestinal epithelium via increased *NPC1L1* expression, and the A allele of rs1761667 decreases CoQ_10_ uptake from blood/tissue fluid into cells by downregulating *CD36* expression; thus predicting the HR to CoQ_10_ supplementation. Notably, the G allele selection of rs2032582 showed a contrasting effect because it may increase the expression of *ABCB1*, resulting in accelerated efflux of molecules from the intestinal epithelium. Rs2032582 (G > T) is in linkage disequilibrium with rs1045642 (C > T) and rs1128503 (C > T), which may alter the higher-order structure of substrate/inhibitor interaction sites, resulting drug-specificity changes [51]. Thus, it may be inferred that ABCB1 translated from the T allele of rs2032582-containing mRNA can efflux more CoQ_10_ molecules than that from the G allele-containing mRNA. Further investigations should address the reason of this discrepancy.

In contrast, in men, none of the eight SNPs was associated with the HR/LR classification (Table 3). This could be attributed to the difference in the number of participants in each sex group; men participants (*n* = 38) were almost half of women participants (*n* = 70). Moreover, the serum CoQ_10_ levels after supplementation and ΔCoQ_10_ values in three men participants (arrowheads in Figure S2) were too high, which were identified as outlier values by the Smirnov–Grubbs test (data not shown). Even when those three men participants, who belonged to both group 1 and group 2, were excluded from the analysis, the serum CoQ_10_ levels before and after supplementation, ΔCoQ_10_, and Δ[CoQ_10_/TC] remained statistically insignificant between the two groups (*p* = 0.21, 0.068, 0.15, and 0.50, respectively; Figure S2B). However, those average values were higher in group 1 than in group 2, giving a possibility that classification based on the four SNPs might be useful for HR/LR prediction in both sexes. Alternatively, gender-specific effects due to estrogen-regulated gene expression could be responsible for the differences observed in response to CoQ_10_ supplementation. Indeed, previous reports showed the gender-related modulation by rs3808607 [52,53], rs2072183 [54,55], rs2032582 [56], and rs1761667 [57]. In either case, whether the classification by the above four SNPs is useful would be clarified by performing the same study with a larger number of men participants.

However, this study has some limitations. First, only eight SNPs from seven genes were evaluated due to throughput limitations and the research budget. These SNPs were carefully selected based on reliable documentation from the PubMed/PMC database from the National Center for Biotechnology Information, USA. However, there are 20, 20, 36, 12, 18, and 178 PubMed-cited SNPs in *SREBP2*, *HMGCR*, *APOB*, *CYP7A1*, *NPC1L1*, and *ABCB1*, respectively (https://www.ncbi.nlm.nih.gov/snp, accessed on 3 January 2021), which may indicate that potentially important SNPs useful for the determination of HR/LR may have been left out form the analysis. Additionally, more suitable SNPs for HR/LR prediction may also exist in other genes. A genome-wide association study with the human SNP array may help find SNPs affecting serum CoQ_10_ levels after long-term supplementation. Second, two types of reduced CoQ_10_ supplements (a soft-encapsulated and a granulated form) were used according to the preferences of the participants. The participants also could switch between supplement types during the intervention. To increase the number of participants and ask them to follow the daily intake protocol of CoQ_10_ supplementation for a long-term period, it was not possible to standardize the administered supplement form. Indeed, around 45% of the participants switched the supplement types during the intervention. Third, it was challenging to monitor the daily supplement intake of the participants for one year. We also provided the participants with a self-check calendar to record the daily intake of the CoQ_10_ supplement when providing the supplements every three months. However, the participants sometimes forgot to record, which made it hard to monitor appropriately. Therefore, all we could do for relevant analyses was that the participants, whose serum ΔCoQ_10_ levels were less than 1 μmol/L, were excluded from the analysis.

Currently, whether these SNPs and the HR/LR prediction are meaningful or useful to obtain conclusive evidence for the beneficial effects of CoQ_10_ remains to be investigated in further detail. A combination of intervention studies with CoQ_10_ and SNPs genotyping would help clarify this issue.

## 5. Conclusions

This study identified four SNPs in *CYP7A1*, *NPC1L1*, *ABCB1*, and *CD36* involved in the regulation of serum CoQ_10_ status after long-term supplementation of reduced CoQ_10_ in women. Classification according to four SNP genotypes could help predict the response to CoQ_10_ supplementations, thereby helping find reliable clinical evidence of the beneficial effects of CoQ_10_ in future interventional studies.

## Figures and Tables

**Table 1:** Sequences of the specific primers and restriction enzymes used for polymerase chain reaction (PCR)-restriction fragment length polymorphism (RFLP) genotyping.

No.	Gene	dbSNP ID	Major Allele	Minor Allele	Forward Primer Sequence	Reverse Primer Sequence	PCR Product Size	Restriction Enzyme	Expected Band Pattern after Digestion (Allele)
1	*SREBP2*	rs133291	C	T	5’-AAC AGT TTG ACA GCA AAG CAG A-3’	5’-CTT TCT CTT GCC CCA TCA TTA C-3’	381 bp	*Btg*I	238 bp + 143 bp (C) 381 bp (T)
2	*HMGCR*	rs3846663	C	T	5’-TCA GCC TAA TCC ATT GTG TCC-3’	5’-CTT TGC ATG CTC CTT GAA CA-3’	333 bp	*Hpy*CH4III	150 bp + 110 bp + 73 bp (C) 183 + 150 bp (T)
3	*APOB*	rs1042034	A	G	5’-TTA TCA AAA GAA GCC CAA GAG G-3’	5’-ACG AAG GGC CAT AAT GTA TTG A-3’	330 bp	*Tsp*RI	330 bp (A)187 bp + 143 bp (G)
4	*CYP7A1*	rs3808607	G	T	5’-AAG GAT GCC ACT GAA AAG AGA C-3’	5’-CTC TCT GGC AAA GCA CCT AAA T-3’	441 bp	*Bsa*I-HF	221 bp + 180 bp + 40 bp (G) 261 bp + 180 bp (T)
5	*NPC1L1*	rs2072183	C	G	5’-AAT GAG TCC CAA GGT GAC CA-3’	5’-ACC ACC GGG ATG ACA GAT AG-3’	362 bp	*Taq*aI	275 bp + 87 bp (C) 362 bp (G)
6	*ABCB1*	rs1045642	C	T	5’-AAA GTG TGC TGG TCC TGA AGT T-3’	5’-TTC TCT TCA CTT CTG GGA GAC C-3’	350 bp	*Mbo*I	172 bp + 152 bp + 26 bp (C) 324 bp + 26 bp (T)
7	*ABCB1*	rs2032582	G	T	5’-ATA GCA AAT CTT GGG ACA GGA A-3’	5’-CCA AGA ACT GGC TTT GCT ACT T-3’	352 bp	*Bse*YI	192 bp + 160 bp (G) 352 bp (T)
8	*CD36*	rs1761667	G	A	5’-GCC TCT GAA TTT ATG CAT GTT G-3’	5’-CGC CTT AGA ATA TTT TGG GAG A-3’	325 bp	*Hha*I	174 bp + 151 bp (G) 325 bp (A)

Abbreviations: dbSNP ID, database single nucleotide polymorphism identifier; PCR, polymerase chain reaction; RFLP, restriction fragment length polymorphism.

**Table 2:** Baseline characteristics of the participants included in the analysis.

Characteristics		All (*n* = 108)	Men (*n* = 38)	Women (*n* = 70)	*p*-Value
Age		64.2 ± 10.4	64.1 ± 10.9	64.2 ± 10.1	0.95
CoQ_10_	(µmol/L)	1.17 ± 0.38	1.28 ± 0.36	1.11 ± 0.38	0.025
TC	(mmol/L)	5.09 ± 0.81	4.87 ± 0.88	5.21 ± 0.75	0.051
CoQ_10_^/^TC	(µmol/mol)	232 ± 74	269 ± 82	213 ± 60	0.0005

Mean ± SD. *p*-values were analyzed by Welch’s *t*-test between men and women.

**Table 3:** Association of single nucleotide polymorphism (SNP) genotypes with high-responder (HR)/ low-responder (LR) classification.

SNP ID	Gene	Major > Minor	*p*-Value (HR/LR)			
			Men		Women	
			ΔCoQ_10_	Δ[CoQ_10_/TC]	ΔCoQ_10_	Δ[CoQ_10_/TC]
rs133291	*SREBP2*	C > T	0.93	0.24	0.19	0.31
rs3846663	*HMGCR*	C > T	0.68	0.061	0.58	0.43
rs1042034	*APOB*	A > G	0.95	0.62	0.80	0.58
rs3808607	*CYP7A1*	G > T	0.41	0.61	0.24	0.037
rs2072183	*NPC1L1*	C > G	0.48	0.46	0.10	0.027
rs1045642	*ABCB1*	C > T	0.32	0.57	0.29	0.27
rs2032582	*ABCB1*	G > T	0.66	0.57	0.049	0.053
rs1761667	*CD36*	G > A	0.13	0.36	0.022	0.075

Abbreviations: ΔCoQ_10_, increased serum coenzyme Q_10_; Δ[CoQ_10_/TC], cholesterol-adjusted increased serum CoQ_10_; HR, high-responder; LR, low-responder; SNP, single nucleotide polymorphism.

**Table 4:** Associations between group 1 and group 2 with HR/LR classification in women.

	N		*p*-Value
	Group 1	Group 2	
ΔCoQ_10_			
More than average (5.05 μmol/L)	19	9	
Less than average	19	23	0.063
Δ[CoQ_10_/TC]			
More than average (952 μmol/mol)	24	9	
Less than average	14	23	0.003

Abbreviations: ΔCoQ_10_, serum coenzyme Q_10_; Δ[CoQ_10_/TC], cholesterol-adjusted serum CoQ_10_; HR, high-responder; LR, low-responder.

## Data Availability

The data that support the findings of this study are available from the corresponding author upon reasonable request.

## Supplementary Materials

The following are available online at https://www.mdpi.com/2076-3921/10/3/431/s1, Figure S1: Genotyping of *SREBP2* rs133291 (a), *HMGCR* rs3846663 (b), *APOB* rs1042034 (c), *CYP7A1* rs3808607 (d), *NPC1L1* rs2072183 (e), *ABCB1* rs1045642 (f) and rs2032582 (g), and *CD36* rs1761667 (h) by PCR-RFLP. Figure S2: Serum levels of CoQ_10_, ΔCoQ_10_, and Δ[CoQ_10_/TC] of group 1 and group 2 in men.
