# Supplementary material for: CYP7A1, NPC1L1, ABCB1, and CD36 Polymorphisms Are Associated with Increased Serum Coenzyme Q10 after Long-Term Supplementation in Women
Source: Antioxidants (Basel). 2021 Mar 11;10(3):431. doi: 10.3390/antiox10030431 (PMC7998724; doi:10.3390/antiox10030431)
Supplement: Supplementary file 1 [file antioxidants-10-00431-s001.zip › Figure S1.pdf]

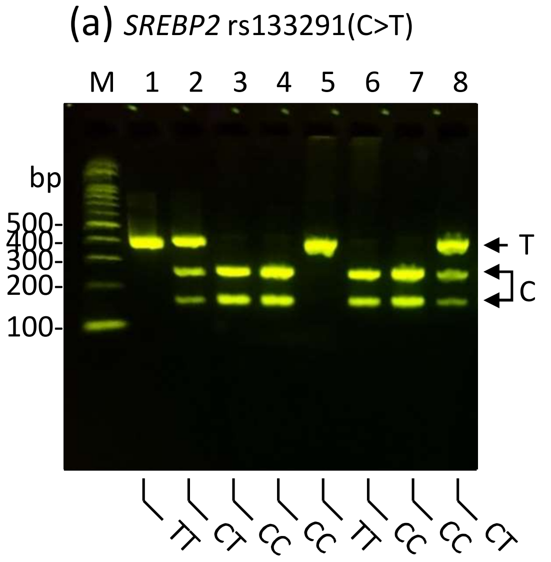

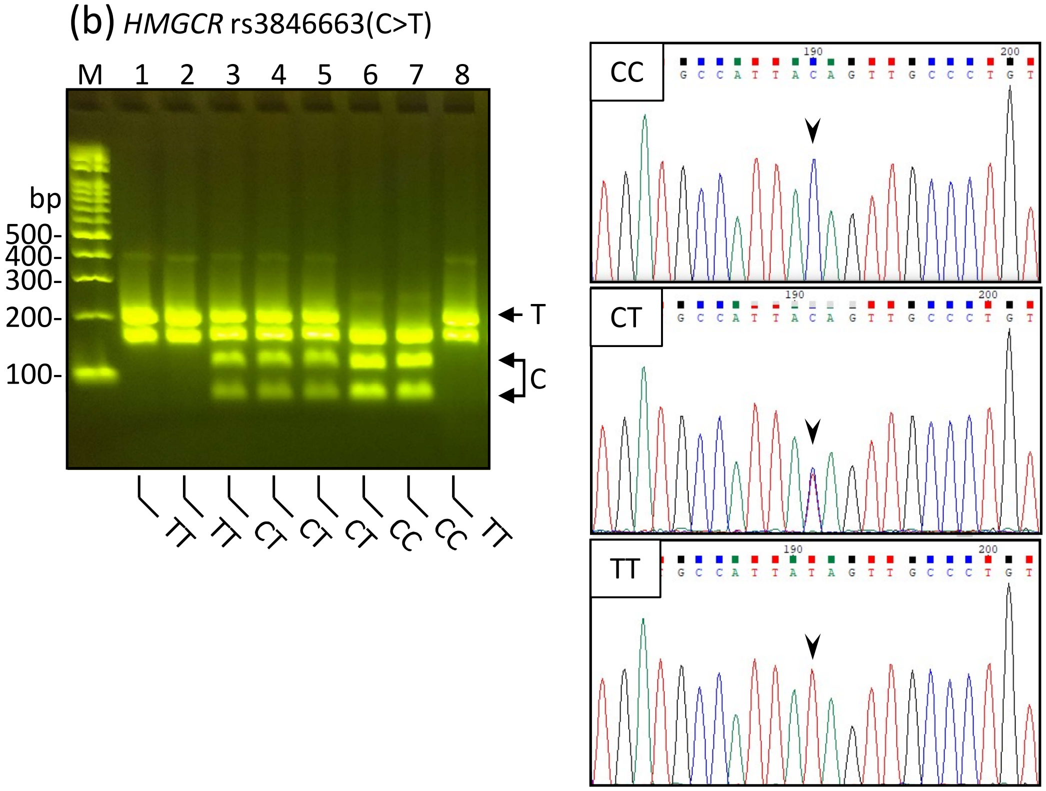

Figure S1. Genotyping of *SREBP2* rs133291 (a), *HMGCR* rs3846663 (b), *APOB* rs1042034 (c), *CYP7A1* rs3808607 (d), *NPC1L1* rs2072183 (e), *ABCB1* rs1045642 (f) and rs2032582 (g), and *CD36* rs1761667 (h) using a PCR-RFLP.

Left panels, examples of electropherogram. Lane M, 100 bp marker ladder; Lanes 1-8, restriction fragments of PCR products. Genotypes determined from the restriction fragments were described under the electropherogram. Right panels, sequencing chromatograms of PCR product. Arrowheads indicate the nucleotide position of the SNPs. DNA sequence data of rs133291 (a) are not available.

(c) *APOB* rs1042034(A>G)

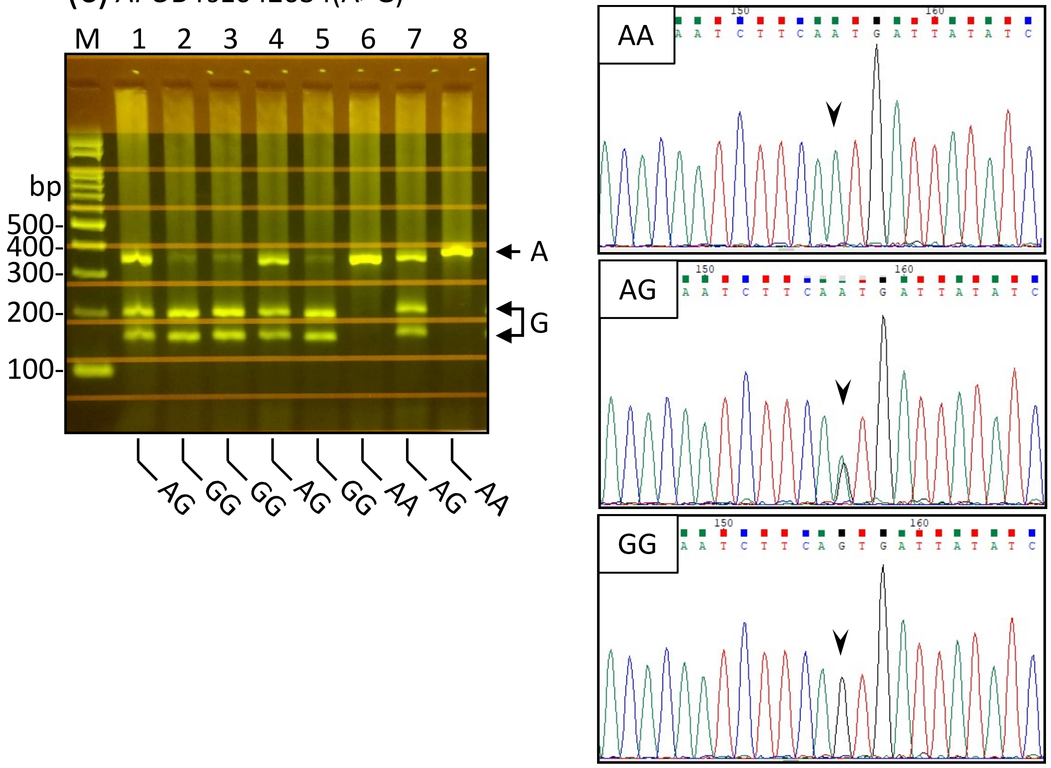

(d) *CYP7A1* rs3808607(G>T)

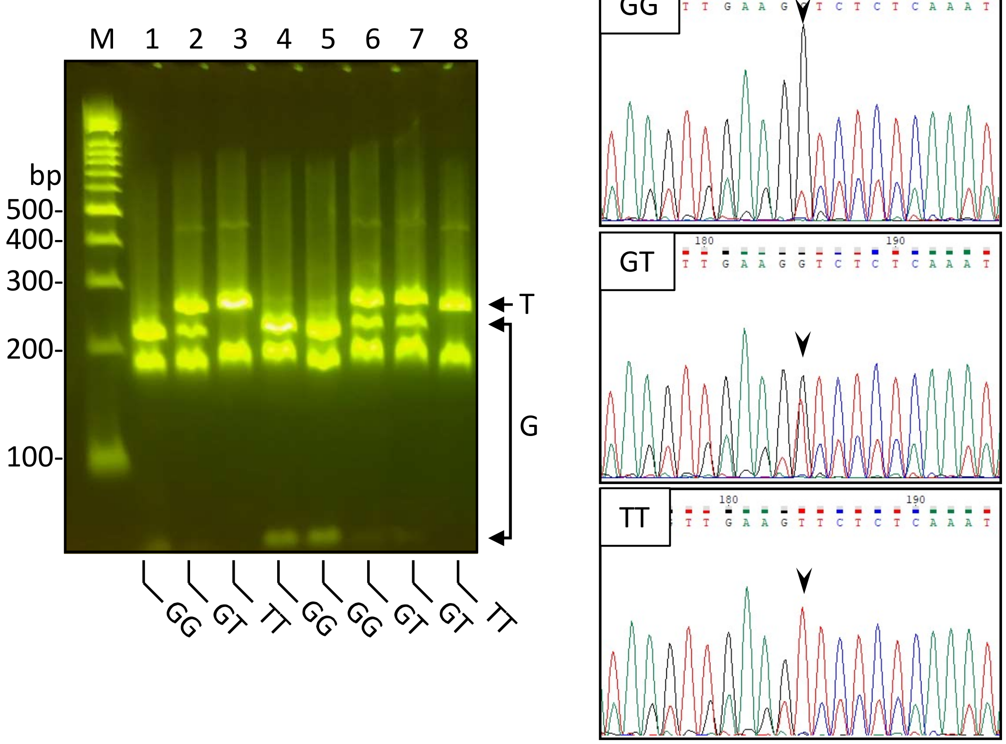

(Figure S1. continued.)

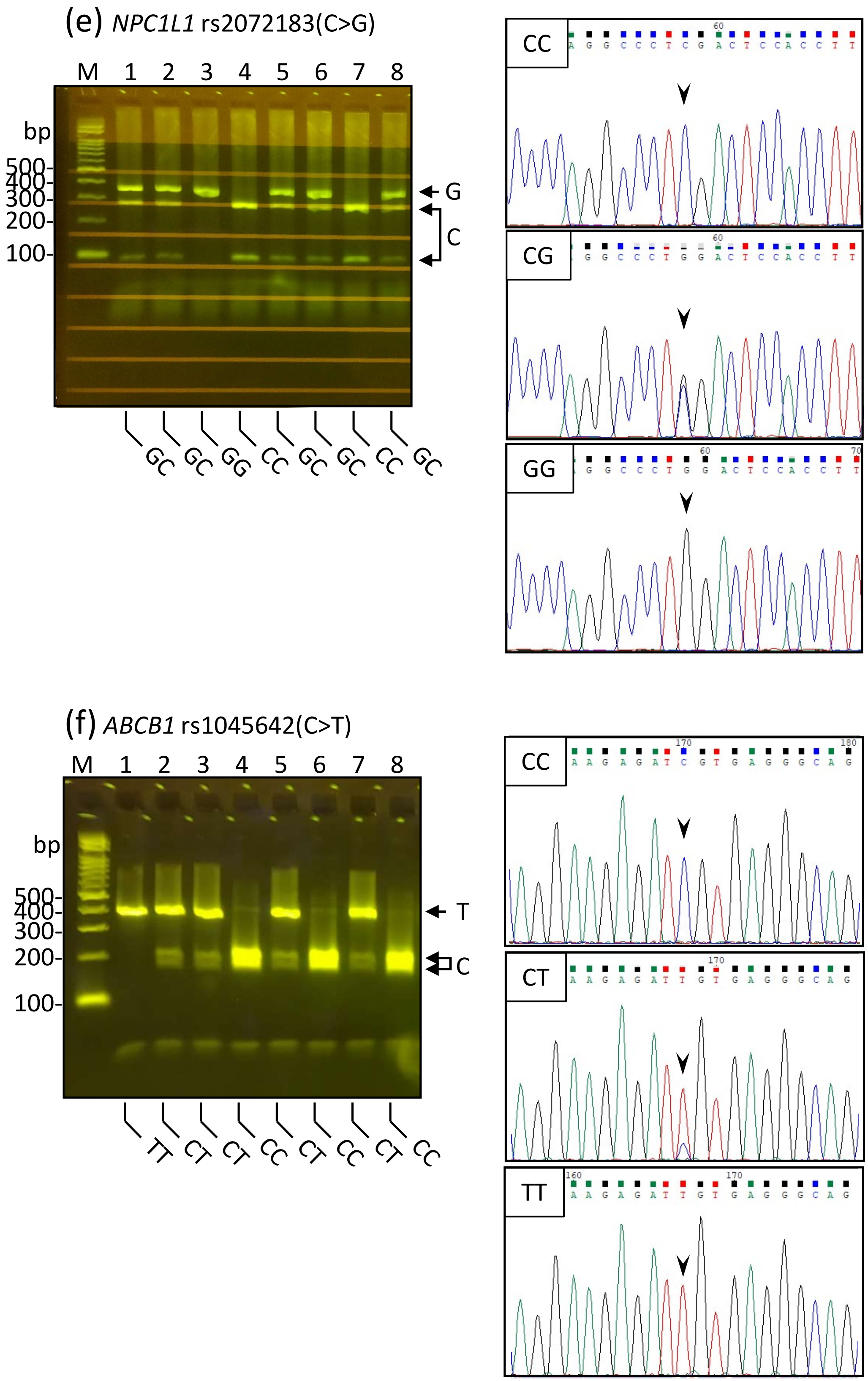

(Figure S1. continued.)

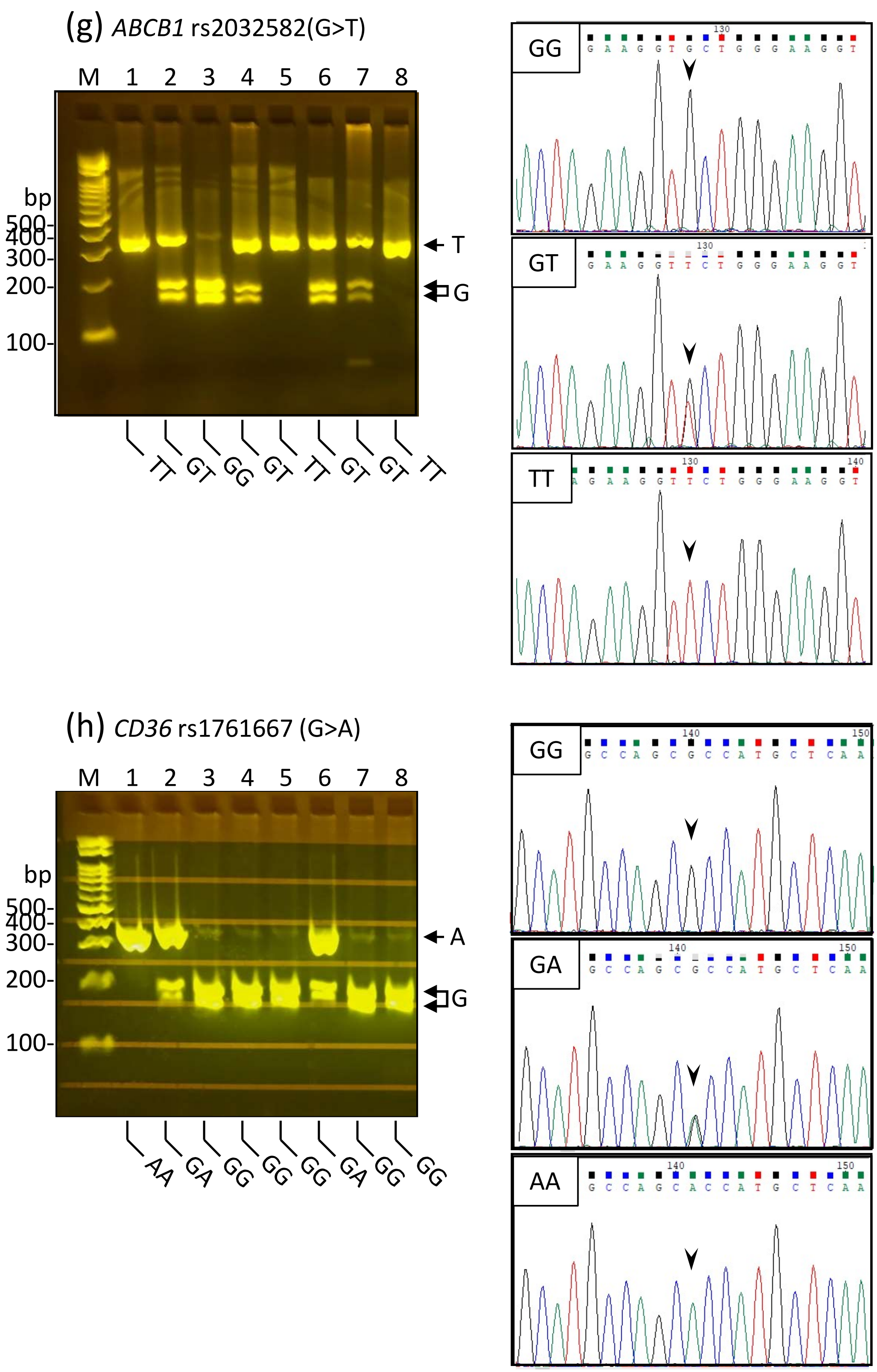

(Figure S1. continued.)
