# Supplementary material for: CYP7A1, NPC1L1, ABCB1, and CD36 Polymorphisms Are Associated with Increased Serum Coenzyme Q10 after Long-Term Supplementation in Women
Source: Antioxidants (Basel). 2021 Mar 11;10(3):431. doi: 10.3390/antiox10030431 (PMC7998724; doi:10.3390/antiox10030431)
Supplement: Supplementary file 1 [file antioxidants-10-00431-s001.zip › Figure S2.pdf]

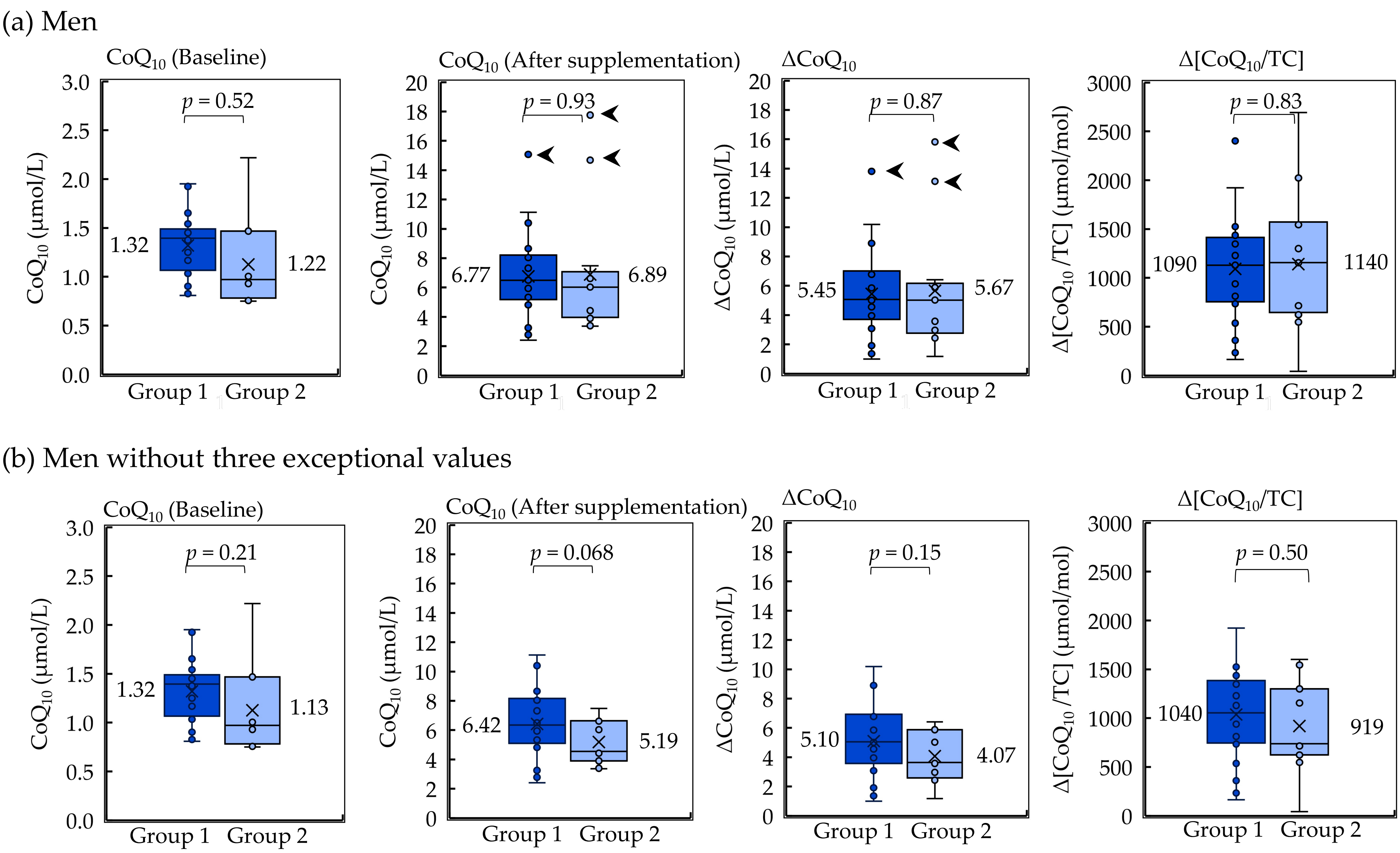

Figure S2. Serum levels of CoQ_{10}, ΔCoQ_{10}, and $\Delta[CoQ_{10}/TC]$ of Group 1 and Group 2 in men.
Box plots for the serum CoQ_{10} levels at baseline and after 1 year of supplementation, ΔCoQ_{10}, and $\Delta[CoQ_{10}/TC]$ for men before (a) and after excluding exceptional values by Smirnov-Grubbs test (b). Arrowheads in (a) indicates exceptional values. The bottom of the box is the 25th percentile, the line that intersects the box is the median, the multiplication sign within the box is the mean, and the top of the box is the 75th percentile. Whiskers above and below the box represent the 10th and 90th percentiles, and the points above and below the whiskers indicate outliers (outwith the 10th and 90th percentiles). No differences between the two groups were observed by Welch's t-test, even after excluding three men with the exceptional values.
